# Relationship between 4-Hydroxynonenal (4-HNE) as Systemic Biomarker of Lipid Peroxidation and Metabolomic Profiling of Patients with Prostate Cancer

**DOI:** 10.3390/biom13010145

**Published:** 2023-01-10

**Authors:** Matea Nikolac Perkovic, Morana Jaganjac, Lidija Milkovic, Tea Horvat, David Rojo, Kamelija Zarkovic, Marijana Ćorić, Tvrtko Hudolin, Georg Waeg, Biserka Orehovec, Neven Zarkovic

**Affiliations:** 1 Division of Molecular Medicine, Ruder Boskovic Institute, HR-10000 Zagreb, Croatia; 2 Centro de Metabolómica y Bioanálisis (CEMBIO), Facultad de Farmacia, Universidad CEU San Pablo, Campus Montepríncipe, ES-28003 Madrid, Spain; 3 Division of Pathology, School of Medicine, University of Zagreb, University Hospital Centre Zagreb, Kispaticeva 12, HR-10000 Zagreb, Croatia; 4 Division of Urology, School of Medicine, University of Zagreb, University Hospital Centre Zagreb, Kispaticeva 12, HR-10000 Zagreb, Croatia; 5 Institute of Molecular Biosciences, Karl Franzens University, A-8010 Graz, Austria; 6 Clinical Hospital Dubrava, HR-10000 Zagreb, Croatia

**Keywords:** cancer, prostate carcinoma, lipid peroxidation, 4-hydroynonenal (4-HNE), metabolomics, GS-MS, LC-MS

## Abstract

An oxidative degradation product of the polyunsaturated fatty acids, 4-hydroxynonenal (4-HNE), is of particular interest in cancer research due to its concentration-dependent pleiotropic activities affecting cellular antioxidants, metabolism, and growth control. Although an increase in oxidative stress and lipid peroxidation was already associated with prostate cancer progression a few decades ago, the knowledge of the involvement of 4-HNE in prostate cancer tumorigenesis is limited. This study investigated the appearance of 4-HNE-protein adducts in prostate cancer tissue by immunohistochemistry using a genuine 4-HNE monoclonal antibody. Plasma samples of the same patients and samples of the healthy controls were also analyzed for the presence of 4-HNE-protein adducts, followed by metabolic profiling using LC-ESI-QTOF-MS and GC-EI-Q-MS. Finally, the analysis of the metabolic pathways affected by 4-HNE was performed. The obtained results revealed the absence of 4-HNE-protein adducts in prostate carcinoma tissue but increased 4-HNE-protein levels in the plasma of these patients. Metabolomics revealed a positive association of different long-chain and medium-chain fatty acids with the presence of prostate cancer. Furthermore, while linoleic acid positively correlated with the levels of 4-HNE-protein adducts in the blood of healthy men, no correlation was obtained for cancer patients indicating altered lipid metabolism in this case. The metabolic pathway of unsaturated fatty acids biosynthesis emerged as significantly affected by 4-HNE. Overall, this is the first study linking 4-HNE adduction to plasma proteins with specific alterations in the plasma metabolome of prostate cancer patients. This study revealed that increased 4-HNE plasma protein adducts could modulate the unsaturated fatty acids biosynthesis pathway. It is yet to be determined if this is a direct result of 4-HNE or whether they are produced by the same underlying mechanisms. Further mechanistic studies are needed to grasp the biological significance of the observed changes in prostate cancer tumorigenesis.

## 1. Introduction

Prostate cancer is the second most frequent malignancy in men worldwide, with more than 1,4 million new cases per year, causing 3.8% of all malignant deaths in 2020 [1]. It is a heterogeneous disease with incidence rates that vary across the world and increase with age. The etiology of prostate cancer is multifactorial and remains largely unknown compared to other common cancers. Some well-established risk factors include advanced age, positive family history, genetic factors, and African ancestry. Prostate cancer is a slow-growing cancer that may be asymptomatic at the early stage and often has an indolent course. Late-stage cancer can present with bone pain, urinary symptoms, and/or weight loss [2].

Most cases of prostate cancer are revealed using digital rectal examination, by diagnostic tests to determine prostate-specific antigen (PSA) levels, transrectal ultrasound (TRUS), and multiparametric MRI, while diagnosis is confirmed by tissue analysis obtained on image-guided transrectal biopsy. Almost all prostate cancers are histologically adenocarcinoma.

The International Society of Urological Pathology (ISUP) consensus conferences in 2005, 2014, and 2019, as well as the 2019 white paper by the Genitourinary Pathology Society (GUPS), report the introduction of computer-aided cancer grading using artificial intelligence [3]. Hence, a series of studies have shown that artificial intelligence-based algorithms can perform prostate cancer grading at the level of international experts in prostate pathology [4].

The tumorigenesis of prostate cancer is accompanied by excessive reactive oxygen species (ROS) production and impaired redox homeostasis. ROS have a dual role in tumorigenesis, and while the moderate elevation of ROS may promote anti-tumor effects, excessive ROS may support the development and progression of cancer [5,6]. Indeed, metastatic prostate cancer cells thrive under excessive ROS, and to achieve their addictive microenvironment, prostate cancer cells regulate their intracellular redox status and respiratory burst of neutrophils [7]. Unsaturated fatty acids are particularly sensitive to ROS-induced injury, and the abstraction of allylic hydrogen initiates lipid peroxidation, yielding reactive aldehydes, such are malondialdehyde, acrolein and 4-hydroxynonenal (4-HNE) [8]. Plasma malondialdehyde [9,10] and the presence of acrolein–protein conjugates in tumor tissues are associated with the progression of prostate carcinoma [11]. However, although the bioactive properties and biological significance of 4-HNE are well-recognized today [12], the possible involvement of 4-HNE in the pathogenesis of prostate cancer is limited, whereas our preliminary findings suggested that 4-HNE is not present in prostate carcinoma cells at all [13].

The biotransformation of free 4-HNE occurs within several minutes, with the maximal catabolism rate varying between tissues [14]. A number of primary and secondary metabolites have been identified, with the predominant urine metabolite being 1,4-dihydroxynonane mercapturic acid [15,16,17]. Moreover, the high reactivity of 4-HNE contributes to its removal via 4-HNE adduction to macromolecules. 4-HNE readily modifies diverse proteins, affecting their structure and function in both physiology and pathology [18,19,20], and is thus often studied in biological tissues and fluids.

Metabolic reprogramming is another hallmark of prostate cancer development and progression. Recent studies have evidenced marked changes in the plasma [21], serum [22], urine [23], and seminal plasma [24] metabolomes of prostate cancer patients. However, whether 4-HNE is linked to these perturbations is unknown. Therefore, in this study, we investigated the presence of 4-HNE-protein adducts in both plasma samples and tissues of prostate cancer patients to search for the possible link between 4-HNE and metabolome perturbation in order to better understand the potential role of 4-HNE in prostate cancer development and its association with metabolic alterations in prostate cancer patients.

## 2. Materials and Methods

### 2.1. Chemicals and Reagents

For metabolomic analyses, the following were used: acetonitrile (ACN) (LC-MS grade, Sigma-Aldrich, Steinheim, Germany), ethanol (EtOH) (LC-MS grade, Sigma-Aldrich, Steinheim, Germany), formic acid (FA) (MS grade, Sigma-Aldrich, Steinheim, Germany), heptane (Sigma-Aldrich, Steinheim, Germany), methanol (MeOH) (Sigma-Aldrich, Steinheim, Germany), O-methoxyamine hydrochloride (Sigma-Aldrich, Steinheim, Germany), N,O-bis(trimethylsilyl) trifluoroacetamide (BSTFA) with 1% trimethylchlorosilane (TMCS) (Pierce Chemical Co., Rockford, IL, USA), and pyridine (Sigma-Aldrich, Steinheim, Germany). Ultrapure water was obtained from the MilliQ®plus185 system (Millipore, Billerica, MA, USA). Tricosane (Sigma-Aldrich, Steinheim, Germany) and 4-chlorophenol (Sigma-Aldrich, Steinheim, Germany) were used as internal standards in the GC-MS analysis. The FAME mix (mix of fatty acid methyl esters; methyl caprylate, methyl caprate, methyl laurate, methyl myristate, methyl palmitate, methyl heptadecanoate, methyl oleate, methyl stearate, methyl eicosanoate, methyl docosanoate) for GC-MS analytical platform was purchased from Supelco (Bellefonte, PA, USA). Ammonium trifluoroacetate (TFA(NH4)), purine, and hexakis(1H,1H,3H-tetrafluoropropoxy)phosphazine (HP) from API-TOF reference mass solution kit (Agilent Technologies, Waldbronn, Germany) were diluted in 95:5 of ACN to water ratio and used as reference solution in the LC-MS analysis.

### 2.2. Subjects and Sample Collection

This study was carried out according to the approval of the Ethic Committee of the University Clinical Hospital Centre Zagreb (approval code 02/21 AG), while each patient signed the informed consent. All of the patients included in this study underwent open radical prostatectomy for localized prostate cancer based on prostate-specific antigen (PSA) values, digital rectal examination (clinical stage ≤ T2c), biopsy findings (≤ Gleason grade group 3), and imaging modalities (computed tomography, bone scintigraphy, magnetic resonance). The blood samples of 30 patients were taken by venipuncture before surgery and collected into ethylenediaminetetraacetic acid (EDTA) tubes with BHT and were centrifuged at 3000× *g* (4 °C) for 20 min to obtain the plasma. The control plasma samples were obtained from 41 fasted, healthy men following the same procedure as in the case of patients. The plasma samples were stored at −80 °C for subsequent analysis.

The surgically obtained tissue specimens were stored in formalin before further evaluation by pathologists, while patients were regularly followed up at the Urology Clinic at the University Hospital Centre Zagreb (approval code 02/21 AG).

### 2.3. Immunohistochemistry

The specimens of the tissue obtained by surgery were fixed in 10% buffered formalin immediately after resection, dehydrated in ethanol, and embedded in paraffin. The representative paraffin blocks of each tumor and surrounding mucosa were cut into three 5µm thin slices and examined by section staining with hematoxylin and eosin and immunohistochemistry using monoclonal antibody for 4-HNE-histidine obtained from the culture medium of the clone derived from a fusion of Sp2-Ag8 myeloma cells with B-cells of a BALBc mouse immunized by HNE-modified keyhole limpet hemocyane [25]. Dilutions of the antibody solution and appropriate reagents from the EnVision detection kit (K 8000, DAKO, Glostrup, Denmark) were used on a DAKO automated immunostainer. The 4-HNE-histidine antigens were localized using enzyme-labeled polymer conjugated to a secondary antibody with 3, 3,-diaminobenzidine as a chromogen and counterstained with hematoxylin (Kemika, Zagreb, Croatia).

The immunohistochemical investigation of the intensity and distribution of 4-HNE in the tumor and the surrounding non-tumorous mucosa was determined by expert pathologists. The presence of 4.HNE-protein adducts in carcinoma cells, stroma, and blood vessels was defined either as negative (0) if there was no immunohistochemical positivity observed or as positive (+) if the presence of 4-HNE-protein adducts was observed. Two pathologists diagnosed each specimen independently.

### 2.4. HNE-ELISA

A previously described in-house protocol [26,27] was used to measure the levels of 4-HNE-protein adducts in the plasma samples of the healthy controls (*n* = 41) and prostate cancer patients (*n* = 30). Briefly, 10 μL of standards/samples (adjusted to 10 mg/mL) per well of an ELISA plate (Nunc Immuno Maxisorp, Thermo Scientific, Nunc A/S, 4000 Roskilde, Denmark) was added into 100 μL of 0.05 M carbonate-binding buffer (pH 9.6; 0.015 M sodium carbonate, 0.035 M sodium bicarbonate) in triplicate and incubated for 5 h at 4 °C. Before the addition of the same monoclonal primary antibody as used for immunohistochemistry and overnight (ON) incubation at 4 °C, the wells were blocked with 5% fat-free dry milk in carbonate-binding buffer) for 3 h at room temperature (RT). The next day, after 30 min of endogenous peroxidase blocking, the wells were incubated with the secondary antibody for 1 h at RT, followed by the addition of the substrate solution and absorbance reading at 450/620 nm. Before each step, the wells were washed five times with a washing buffer. Concentrations of 4-HNE protein adducts were interpolated from the standard curve and expressed as pmol 4-HNE protein adducts/mg of proteins.

### 2.5. Sample Preparation and Metabolite Extraction

#### 2.5.1. LC-MS Platform

On the day of analysis, the plasma samples (stored at −80 °C) were slowly defrosted on ice and vortex-mixed for 2 min. For metabolite extraction, 100 µL of each sample was mixed with cold MeOH:EtOH (1:1 ratio), vortex-mixed for 2 min and incubated on ice for 5 min. After centrifugation (16,000× *g*, 10 min, 4 °C), the remaining supernatant (200 µL) was transferred to crimp-top clear glass vials with a 300 µL insert for further LC-MS analysis.

#### 2.5.2. GC-MS Platform

For the GC-MS analysis, the samples were aliquoted on the same day as the LC-MS analysis. The deproteinization was achieved by mixing the plasma samples (100 µL) with 300 µL of cold ACN. The samples were vortex-mixed for 2 min and incubated on ice for 5 min. The corresponding aliquot of each sample (100 µL) was centrifugated (16,000× *g*, 10 min, 4 °C), and 100 µL of the supernatant was transferred to the crimp-top clear glass vials with the insert, and subsequently, 20 µL of 4-chlorophenol (100 ppm, in ACN) was added. The samples were evaporated to dryness using a Speedvac Concentrator (Thermo Fisher Scientific, Waltham, MA, USA). Methoximation was performed by adding 10 μL of O-methoxyamine hydrochloride (15 mg/mL in pyridine). The samples were vigorously vortex-mixed for 5 min, followed by three cycles of ultrasonication (2 min) and vortex mixing (2 min). In the next step, the vials were incubated in darkness at room temperature for 16 h. The next day, 10 μL of BSTFA with 1% TMCS was added to each vial for silylation, and the samples were vortex-mixed for 5 min. Silylation was carried out at 70 °C for 1 h. After the samples cooled down to room temperature, 100 μL of tricosane (20 ppm in heptane) was added as an internal standard, and the samples were vortex-mixed for 2 min. Six blank samples (ACN to water ratio 3:1) were prepared in the same way as the plasma samples (including deproteinization and derivatization steps).

### 2.6. Preparation of Quality Control Samples (QCs)

The QCs were prepared separately for the LC-MS and GC-MS platforms. They are necessary to equilibrate/stabilize the system before analyzing the actual set of samples and, afterward, to monitor system stability and reproducibility during the analysis. Individual QCs were prepared by pooling and mixing equal volumes of each plasma sample (10 µL). They were processed in the same way as the actual samples following all of the previously described steps.

### 2.7. Metabolomics Analysis

To achieve a wider metabolite coverage, all of the samples were analyzed using two complementary analytical platforms, LC-ESI-QTOF-MS (abbreviated as LC-MS) and GC-EI-Q-MS (abbreviated as GC-MS).

#### 2.7.1. Fingerprinting by LC-ESI-QTOF-MS

The liquid chromatography system, Agilent Technologies Series 1200 binary solvent delivery system (Agilent Technologies, Waldbronn, Germany), comprised of a binary pump, an integrated degasser, and an autosampler with a thermostat, coupled to an Agilent 6520 Accurate-Mass Q-TOF detector, was used to analyze the metabolic profile of the samples. For each sample, a volume of 10 µL has been injected into a reversed-phase column (Discovery® HS C18 HPLC Column, 515 cm × 2.1 mm, 3 µm; Supelco, Bellefonte, PA, USA) with a pre-column (Discovery® HS C18 HPLC Column, 2 cm × 2.1 mm, 3 µm; Supelco, Bellefonte, PA, USA), which was kept at 60 °C during the analysis. The elution conditions employed a flow rate of 0.6 mL/min with a gradient of solvent A (H2O with 0.1% FA) and solvent B (ACN with 0.1% FA). The analysis started with 25% of the mobile phase B and then increased to 95% of B in a time period of 35 min (0–35 min). The gradient then returned to the initial conditions in 1 min time (35–36 min), 25% of the mobile phase B, and these conditions were maintained until the end of the analysis (36–45 min). All of the samples were analyzed in both positive and negative ESI modes (full-scan ranging from 50 to 1000 *m*/*z*), with a scan rate of 1.02 scans/s. Two reference masses were continuously infused during the entire duration of the analysis to ensure a constant mass correction: 121.0509 (purine, detected *m*/*z* [C_5_H_4_N_4_+H]^+^) and 922.0098 (HP, detected *m*/*z* [C_18_H_18_O_6_N_3_P_3_F_24_+H]^+^) for the positive mode, and 112.9855 (TFA(NH_4_), detected *m*/*z* [C_2_O_2_F_3_(NH_4_)-H]^−^) and 966.0007 (HP+FA, detected *m*/*z* [C_18_H_18_O_6_N_3_P_3_F_24_+FA-H]^−^) for the negative mode.

Tandem mass spectrometry (MS/MS) was performed to facilitate the identification of significant metabolites using the same LC (Agilent 1200)-QTOF-MS (Agilent 6520) platform and the same chromatographic conditions as applied for the primary LC-MS analysis. The selected ions were targeted for fragmentation by collision-induced dissociation (CID) based on the previously determined accurate mass and retention time. Multiple collision energies (10 eV, 20 eV, and 40 eV) were used. The identity of the compounds was confirmed by comparing the fragmentation pattern of the selected ion with a public library of MS/MS spectra.

#### 2.7.2. Fingerprinting by GC-EI-Q-MS

The Agilent 7890A gas chromatograph, with an autosampler (Agilent Technologies 7693), coupled to an inert MSD with Quadrupole (Agilent Technologies 5975) was used for the metabolomic fingerprinting of the plasma samples. For each derivatized sample, a volume of 2 μL was injected, with a split ratio of 1:10, into a Restek 20,782 deactivated glass–wool split liner. The compounds were separated using the GC-Column DB-5MS (length: 30 m, internal diameter: 0.25 mm, film thickness: 0.25 μm, packing: 95% dimethylpolysiloxane/5% diphenylpolysiloxane) with a pre-column (10 m J&W integrated with Agilent 122-5532G). The constant flow rate of the helium carrier gas was set to 1 mL/min, and the injector temperature was held at 250 °C. The temperature of the column oven was set at 60 °C (held for 1 min), with an increased rate of 10 °C/min until the temperature reached 325 °C. This temperature was maintained for up to 10 min before the injection of the next sample. The detector transfer line was set at 290 °C, while the filament source and quadrupole temperatures were set at 230 °C and 150 °C, respectively. The total analysis for each sample lasted 37.5 min. The electron ionization (EI) energy was set to 70 eV. The system collected the mass spectra in a mass range between 50 and 600 *m*/*z* at a rate of 2 spectra/s.

### 2.8. Data Treatment

#### 2.8.1. LC-MS Data Treatment

The data treatment was done using Agilent MassHunter software tool (Agilent Technologies, Waldbronn, Germany). Firstly, the quality of the analysis for all of the analyzed samples and QCs was checked by inspecting and assessing the quality of the total ion chromatograms (TIC), checking the pressure curves to assess the stability of chromatographic conditions, and reviewing the stability of the reference masses’ signal (using Agilent MassHunter Quantitative Analysis software, version B.07.00). All of the samples have passed all of the checkpoints. Subsequently, the raw data were imported into Agilent MassHunter Profinder software (version B.08.00) for deconvolution. The Molecular Feature Extraction (MFE) algorithm was used for deconvolution, creating a list of possible molecular features that matches a gaussian distribution of co-eluting ions related by charge-state, isotopic distribution and/or the presence of different adducts, and dimmers. Subsequently, a second deconvolution step was performed using the Recursive Feature Extraction (RFE) algorithm, which re-integrates the MFE results, improving the quality of the final features list. The resulting list of statistically significant accurate masses was annotated using the CEU Mass Mediator [28] search tool (maximum error mass ± 20 ppm) in order to assign tentative metabolite candidates. The matched compounds were identified using the accurate mass and by checking their isotopic pattern. Only the features with the highest score were kept for further identity confirmation. The biological role of each suggested compound was additionally evaluated to exclude the unrelated and impossible identification matches. Tandem mass spectrometry (MS/MS) was performed for the statistically significant annotated features in positive and negative ionization. The final identification of these compounds was performed by matching the fragmentation spectra as described by Naz and colleagues [29], using different databases such as HMDB [30], METLIN [31], KEGG [32], and LipidMaps [33]. For the identification of the compound, we considered proper retention time, accuracy mass (maximum error mass ± 20 ppm), and at least two MS/MS fragments.

#### 2.8.2. GC-MS Data Treatment

The data treatment was done using Agilent MassHunter software tool (Agilent Technologies, Waldbronn, Germany). After inspecting and assessing the quality of the total ion chromatograms (TIC) for all of the analyzed samples, QCs, and blanks (using Agilent MassHunter Quantitative Analysis software, version B.07.00), and after checking the reproducibility of the signals of the internal standards (4-chlorophenol for derivatization and tricosane for analytical performance), all the samples were accepted. Then, the raw data files were imported into the Agilent MassHunter Unknowns Analysis software (version B.09.00) for deconvolution and identification using targeted libraries, Fiehn library, (version 2013, UC Davis, Davis, CA, USA) and the in-house CEMBIO spectral library for plasma samples. The identities of the compounds were assigned based on the retention time (RT) and mass spectra. The next step was an additional checking of the identified compounds and the non-identify features using the NIST library (National Institute of Standards and Technology, library 2.2 version 2014, Gaithersburg, MD, USA). The alignment of the obtained data was performed with the help of Agilent MassProfiler Professional software (version 13.0) and exported into Agilent MassHunter Quantitative Analysis (version B.09.00) for peak integration. The abundance of each compound in the obtained data matrix was normalized according to the IS (tricosane) abundance, and the blank subtraction was performed prior to statistical analysis.

### 2.9. Statistical Analysis

For both LC-MS and GC-MS, before the statistical analysis of the obtained data, the raw variables were filtered based on the criteria proposed by Godzien and colleagues [34]. The variables retained were (i) present in ≥80% of the QCs (with relative standard deviation (RSD) <30% in QC samples); or 9ii) present in <20% of the QCs, but present in ≥50% of the samples in a specific subject group. Both sets (i and ii) were subsequently modeled. To correct for the intra-batch effect, the analytical variations in QC samples were assessed, and the Quality Control-Robust Spline Correction (QC-RSC) algorithm was used when necessary, as suggested by Kuligowski and colleagues [35]. Support vector regression (QC-RSC) was performed using MATLAB (7.10.0.499, MathWorks, Natick, MA, USA) and the LIBSVM library [36] (software available at http://www.csie.ntu.edu.tw/~cjlin/libsvm). After removing intra-batch effects, normalization was used to decrease the unwanted variations arising from errors in the sample preparation [37]. Auto Scaling (Unit Variance Scaling, UV) was used to normalize and scale metabolic signals based on the standard deviation of metabolomics data [38]. In the case of the GC-MS data, additionally, the abundance of all of the detected compounds was normalized by the signal of internal standard (tricosane) in each sample. The missing values in the data sets were replaced with zeros.

The SIMCA-P+ software (version 15.0.2.5959, Umetrics, Umea, Sweden) was used for multivariate statistical analyses, including building up Principal Component Analysis (PCA) models and Orthogonal PLS-DA (OPLS-DA) models. Based on OPLS-DA models, variable importance in the projection (VIP) values were obtained. Variables with VIP > 1.00 were considered significant. The MetaboAnalyst 5.0 software was used for metabolomics data pathway analysis [39,40].

Univariate statistical analysis was carried out using MATLAB (7.10.0.499, MathWorks, Natick, MA, USA) or GraphPad Prism software (GraphPad Software, San Diego, CA 92108, USA). The Shapiro–Wilk test was used to check for normal distribution in the data set. For the comparison of metabolite abundances between groups, Student’s t-test or Mann–Whitney U test was performed, depending on the distribution of the data, followed by Benjamini–Hochberg (FDR, false discovery rate) post hoc correction for multiple comparisons. An unpaired t-test with Welch’s correction was used to detect the differences in the levels of 4-HNE protein adducts between healthy controls and prostate cancer patients. The relationship between significantly altered metabolites and levels of plasma 4-HNE-modified proteins in both cancer patients and healthy subjects was tested using Spearman’s correlation coefficient.

All of the tests were two-tailed, and *p* < 0.050 was considered significant. In the case of metabolomic data, metabolites with significantly different abundance between the two subject groups were determined by the combination of multivariate and univariate statistics.

Fold change was computed as the ratio between the mean metabolite abundance in the PC group cohort relative to the healthy control group. The percentage of change (%) was calculated as follows: [(average value in the PC group − average value in the control group)/(average value in the control group)] × 100, with positive values indicating increased abundance and negative values decreased abundance of specific metabolites in the PC group when compared to the healthy control group.

## 3. Results

**Figure 1:**
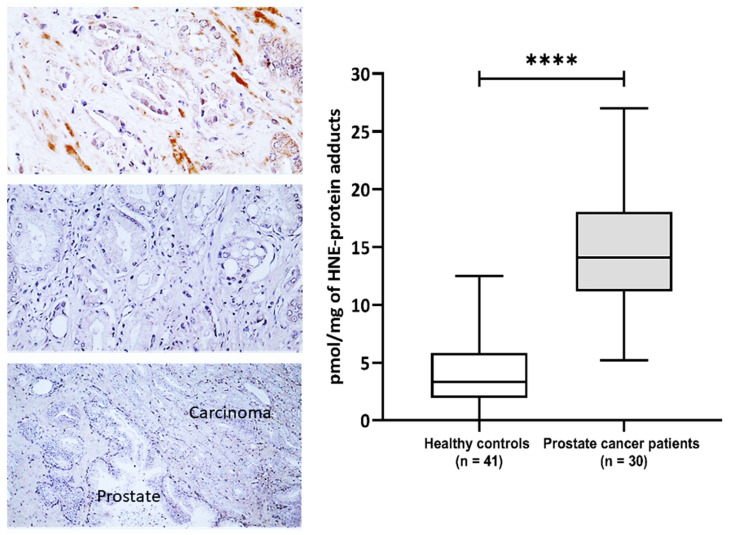
**The 4-hydroxynonenal (4-HNE) modification of plasma and prostate cancer tissue proteins.** Left—Immunohistochemistry of prostate carcinoma specimens obtained by monoclonal antibody specific for the 4-HNE-protein adducts visualized the 4-HNE presence by brown color (DAB staining), while negative cells were contrast-stained blue by hematoxylin. Photo on the top shows rare immunopositivity of the stromal cells observed only for one patient/cancer (200×), the middle photo shows the most usually observed absence of 4-HNE in cancer and in stromal cells (200×), while the lower photo shows the absence of 4-HNE both in cancer and in the adjacent prostate tissue (100×). Right—Plasma concentration of 4-HNE–protein adducts (pmol/mg of protein) in samples of healthy controls (*n* = 41) and prostate cancer patients (*n* = 30). Results are presented as a box and whisker plot. The line in the box represents the median, while the interquartile range (IQR) box represents the middle quartiles (the 75th minus the 25th percentile). The whiskers on either side of the IQR box represent the lowest and highest quartiles of the data. The ends of the whiskers represent the maximum and minimum of the data. **** *p* < 0.0001.

This study included 30 patients with pathohistologically verified prostate cancer and 41 healthy male controls aiming to evaluate the impact of 4-HNE on plasma proteins and metabolome. The general characteristics of the prostate cancer patients are presented in Supplementary Table S1. The prostate-specific antigen (PSA) values before surgery ranged from 3.5 to 41 with a median value of 6.25, while the median of Gleason values of tumor differentiation was 7 (range 6–8) (Supplementary Table S1). The immunohistochemical appearance of 4-HNE-protein conjugates is shown in (Figure 1). The 4-HNE-modified proteins were not detected in any cancer cell, while the presence of 4-HNE-modified proteins was detected only in the stromal cells and blood vessels in cancer tissue of only one patient (patient no.1). The results of the immunohistochemical findings for each prostate cancer tissue are included in Supplementary Table S1. The comparison of 4-HNE-positive staining in cancer and adjacent tumor tissue also did not reveal any significant differences, i.e., 4-HNE was neither present in cancer nor in non-malignant surrounding prostate tissue. However, a significant increase in 4-HNE-modified proteins was observed in the plasma of patients with prostate cancer (Figure 1). Hence, the plasma samples of prostate cancer patients have significantly more 4-HNE-protein adducts compared to the healthy controls (*p* < 0.0001). The levels of 4-HNE-protein adducts did not correlate with the PSA values.

**Figure 2:**
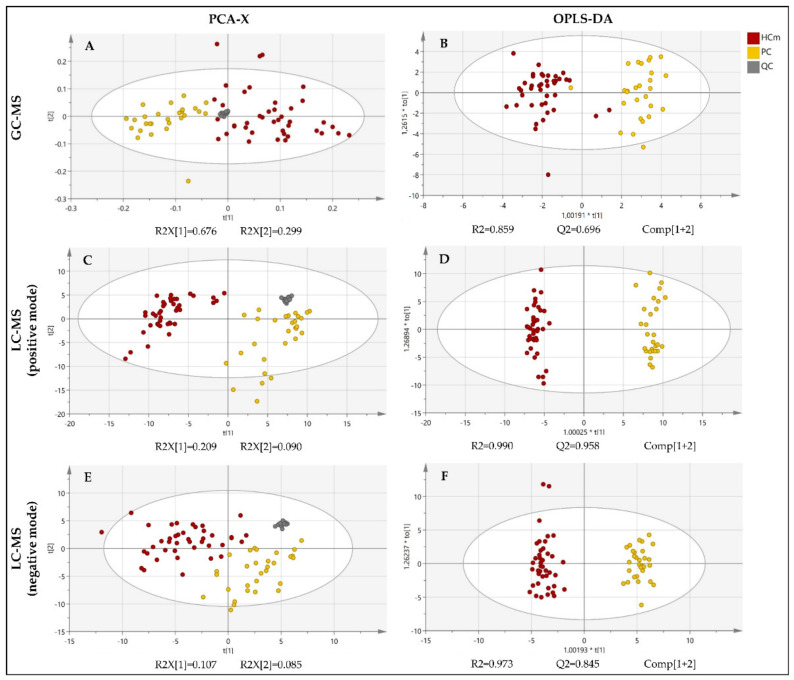
PCA and OPLS-DA score plot of the untargeted metabolomics analysis of plasma samples from patients diagnosed with prostate cancer (PC) and healthy male control subjects (HCm). Plots were obtained using SIMCA-P+ software (version 15.0.2.5959, Umetrics, Umea, Sweden). (**A**) PCA score plot for the GC-MS analysis; (**B**) OPLS-DA score plot fort the GC-MS analysis; (**C**) PCA score plot fort the LC-MS(+) analysis; (**D**) OPLS-DA score plot fort the LC-MS(+) analysis; (**E**) PCA score plot fort the LC-MS(−) analysis; (**F**) OPLS-DA score plot fort the LC-MS(−) analysis; QC = quality control. t[1]: the first principal component, t[2]: the second principal component, to[1]: the first orthogonal component, *: multiplication sign.

The fingerprinting of the plasma metabolome revealed distinct metabolic signatures of prostate cancer patients and healthy controls (Figure 2). To explore the differences in the overall metabolomic profiles of the prostate cancer patients and healthy control subjects, the PCA of all of the samples was used. The PCA showed a clear distribution between the two groups of subjects. Note that the PCA is a non-supervised multivariant model in which the tight cluster of the QCs validates the analytical performance and the clinical model, corroborating the biological differences between the clinical groups. Subsequently, a supervised OPLS-DA model and the VIP score were used in order to identify the metabolites that contributed the most to the difference between patients with prostate cancer and healthy subjects.

The PCA score plots revealed a clear and separate clustering between two groups of subjects (Figure 2). All of the OPLS-DA models were built from one predictive component and two orthogonal components (Figure 1). For all of the OPLS-DA models, the R2(cum) and Q2(cum) values exceeded 0.5, indicating the robustness of the models. This suggests that the models fit the data very well and have a good predictive ability. All three OPLS-DA models were evaluated with permutation analysis (Figure S1A). The permutation analyses strongly indicate that the original models are valid since the criteria for validity are satisfied (all permutated Q2-values to the left are lower than the original points to the right, and the blue regression line of the Q2-points intersects the vertical axis below zero) (Figure S1A). All of the permutated R2-values and Q2-values are lower than the original values on the right (Figure S1A). To identify strong and moderate outliers, we used Hoteling’s T2 line plot (Figure S1B) and the DModX test (Figure S1C). In the case of GC-MS, an LC-MS(+), we detected one outlier in each data set (Figure S1B,C). In the case of GC-MS, the outlier was one participant from the healthy control group, and in the case of LC-MS(+), it was one participant with prostate cancer.

Using the GC-MS approach, a total of 18 metabolites were found to be significantly altered between patients with prostate cancer and healthy control subjects (Table 1). These differential metabolites were mainly fatty acyls, organic acids and their derivates, or different carbohydrates and carbohydrate conjugates. The list of metabolites with significantly different levels between the cancer patients and healthy men was determined by the combination of multivariate and univariate statistics, as presented in Table 1.

Thus, the obtained results indicate increased levels of different fatty acids in patients with prostate cancer compared to the healthy controls, except for caproic acid, the level of which was significantly higher in the control subjects (Table 1). All carbohydrates were increased in prostate cancer, and the same trend was observed for cholesterol (Table 1). The analysis of the organic acids indicated elevated levels of 2-hydroxybutyric, 3-hydroxybutyric, and 2-ketoisocaproic acid and decreased levels of lactic and pyruvic acid in prostate cancer compared with healthy men (Table 1). It should be noted that the elevation of different fatty acids may indicate a catabolic shift of metabolism due to cancer.

The LC-MS metabolite profiling of the plasma samples from the prostate cancer patients resulted in large spectra of metabolites. To explore the differences in the overall metabolomic profiles of the prostate cancer patients and healthy control subjects, obtained by both positive and negative ionization modes, the PCA of all of the samples was used. The PCA showed a clear distribution between the two groups of subjects (Figure 2). A supervised OPLS-DA model and the VIP score were used to identify the metabolites that contributed the most to the difference between patients with prostate cancer and healthy subjects. The LC-MS followed an analogous data treatment, as described for GC-MS. The resulting list of compounds is presented in Table 2. The compounds that could not be identified according to their MS/MS spectra were excluded.

The metabolites that were shown to be significantly altered in the prostate cancer patients compared to the control subjects were mainly fatty acyls, glycerolipids, or organic acids and their derivates (Table 2). Notably, increased levels of fatty acyls, including different fatty acids, docosapentaenoic acid (docosanoid), 9-HODE (9-hydroxyoctadecadienoic acid), octadecadienal (fatty aldehyde), and fatty acid ester tetradecenoylcarnitine, were found in patients with prostate cancer when compared to healthy controls, except for one fatty aldehyde, decadienal, whose amount was significantly higher in the control subjects (Table 2).

The abundance of several monoacylglycerols was also higher in prostate cancer patients when compared to healthy subjects (Table 2). The levels of arginine, threonylhistidine, biliverdin, retinal, and pregnenolone were significantly increased in the plasma of cancer patients, while O-methoxycatechol-O-sulphate, pyrocatechol sulfate, and hyodeoxycholic acid had lower levels in prostate cancer when compared to the control group (Table 2).

The potential impact of elevated amounts of 4-HNE protein adducts in the plasma samples on plasma metabolome was further examined. Hence, correlation analysis of metabolites significantly altered in prostate cancer patients with levels of plasma 4-HNE-modified proteins in both cancer patients and healthy men samples is presented in Table 3.

Plasma caproic acid, linoleic acid, stearic acid, and pyruvic acid showed significant correlations with the plasma levels of 4-HNE protein conjugates in healthy control samples. Stearic and linoleic acid correlated positively with the level of 4-HNE protein conjugates in healthy controls, although the strength of the correlation was moderate and weak, respectively. Contrary, in the same samples, a weak negative correlation was observed for caproic and pyruvic acid.

Only plasma linoleic acid, oleic acid, stearic acid, and pyruvic acid showed significant correlations with the plasma levels of 4-HNE protein conjugates in healthy control samples. For linoleic acid, oleic acid, and stearic acid, we observed a moderate positive correlation with the levels of 4-HNE protein conjugates, while pyruvic acid had a moderate negative correlation in the healthy controls. While all of these correlations were lost if prostate cancer was present, in the plasma samples of prostate cancer patients, new and significant correlations were observed between plasma 4-HNE protein conjugates and plasma metabolites 2-ketoisocaproic acid, 9-HODE, eicosapentaenoic acid, hexadecanedioic acid, methyl hexadecanoic acid, MG(18:2(9,12)), octadecadienal (9,12), palmitoleic acid, pregnenolone, and retinal.

A strong positive correlation between 4-HNE protein adducts and plasma metabolites was recorded for 9-HODE and pregnenolone, while a moderate positive correlation was observed for eicosapentaenoic acid, methyl hexadecanoic acid, MG(18:2(9.12)), and octadecadienal (9.12) and retinal. Furthermore, a significant but weak positive correlation was observed for 2-ketoisocaproic acid. On the contrary, a moderate negative correlation was found for 4-HNE and hexadecanedioic and palmitoleic acid.

Finally, to identify the pathways potentially affected by 4-HNE that could explain the observed changes, we further performed pathway analysis using MetaboAnalyst 5.0 to find that only the pathway of unsaturated fatty acids biosynthesis emerged as significant (*p* = 0.000366, FDR = 0.0308).

## 4. Discussion

The development and progression of cancer are closely associated with oxidative stress that may result in the peroxidation of lipids yielding 4-HNE. The presence of 4-HNE has been implicated in the tumorigenesis of diverse types of cancers, and nowadays, it is well recognized that the occurrence of 4-HNE in serum/plasma samples as well as in tumor and adjacent tumor tissue is tumor-type-specific [12]. However, this is the first study linking elevated 4-HNE-protein conjugates present in the plasma of prostate cancer samples with altered metabolome.

Interestingly, although a significant increase in 4-HNE levels was observed in the plasma samples of cancer patients, 4-HNE was not detected in the tumor tissue or in the adjacent normal tissue, unlike the previously revealed increase in 4-HNE in non-malignant cells surrounding different kinds of cancer tissue [8,41,42,43]. This could be attributed to induced transcription factor NF-E2-related factor 2 (Nrf2), which is activated in prostate cancer independently of ROS [44] and regulates 4-HNE metabolism and activity [45]. The removal of 4-HNE, especially its protein adducts, from cancer cells might be crucial for their survival because the proapoptotic effects of 4-HNE are associated with the aldehyde’s binding to the cellular proteins, especially in cancer cells that are more sensitive to the toxic effect of 4-HNE than are normal, non-malignant cells [46,47,48].

Perhaps another reason why 4-HNE was detected neither in prostate carcinoma cells nor in non-malignant cells may be acrolein, another aldehydic product of lipid peroxidation, which can activate (similar to 4-HNE) the Nrf2 [49]. Namely, acrolein was found to be abundant in the malignant and stromal cells of prostate carcinoma tissues [11], as well as in benign and malignant colon neoplasms and in non-malignant tissue in the vicinity of tumors [50]. However, in the prostate tissue, acrolein could originate not only from oxidized lipids but also from protamines, such as spermine and spermidine, which are abundant in the prostate and are involved in the regulation of cellular proliferation and differentiation, again similar to 4-HNE [48,51]. Moreover, similar to 4-HNE, acrolein can be involved in inflammatory signaling and eventually even in spontaneous cancer regression [5,6,52,53,54]. Therefore, future studies should evaluate this option, too.

On the other hand, the absence of 4-HNE in prostate carcinoma tissue associated with the increase in 4-HNE in the blood of patients with prostate cancer might reflect the elimination of the aldehyde from the carcinoma cells into the blood or enhanced synthesis and release of 4-HNE from remote tissues, notably blood vessels, as was observed in the case of the visceral adipose tissue of people with metabolic syndrome and in COVID-19 patients due to oxidative vascular stress [55,56,57]. However, in both cases, the 4-HNE-protein adducts accumulated within the affected tissues, which was not the case with prostate carcinoma. Therefore, it is more likely that increased 4-HNE levels reflected systemic alteration of the lipid metabolism and oxidative stress in patients with prostate cancer, associated with the onset of cancer development. Since the control group in our study did not comprise patients but healthy men (mostly hospital crew volunteers) who did not have any records or symptoms of prostate or any other organ illness that would jeopardize their consideration as healthy persons, we did not collect their specific baseline characteristics. Therefore, further studies should consider deeper comparisons of the baseline characteristics of patients and control subjects to evaluate their possible differences and relevance for the findings obtained by metabolomics and specific 4-HNE-ELISA. Similarly, it would be interesting to repeat the analyses using the samples of blood of patients after a prolonged time after surgery, i.e., after complete recovery, to compare these with the results obtained before surgery. These are all goals of our further work.

The metabolomic profiling of the plasma samples obtained from patients with prostate cancer, performed using the combined GC-MS and LC-MS approach, revealed a significantly altered abundance of different fatty acids, organic acids, glycerolipids, carbohydrates, and sterol lipids in prostate cancer patients when compared to the healthy men controls. Using GC-MS, we detected and identified 40 compounds, out of which 18 were found to be differentially present in prostate cancer patients in comparison to the healthy control. The LC-MS analysis, in positive and negative ionization mode, was also applied for the identification of metabolites that were significantly altered between two groups of subjects. Finally, we annotated 86 metabolites and confirmed the identity of 23 of them by using their exact mass, retention time, and specific MS/MS fragmentation patterns.

Our study suggests a positive association of different long-chain fatty acids (palmitoleic acid, linoleic acid, oleic acid, eicosapentaenoic acid (EPA), methylhexadecenoic acid, hexadecanedioic acid, docosapentaenoic acid (9-HODE) and medium-chain fatty acids (caprylic acid, lauric acid, palmitic acid, stearic acid) with a prostate cancer diagnosis. Only caproic acid levels were negatively related to prostate cancer diagnosis. Other studies have already suggested a possible relationship between blood fatty acid composition and the risk of prostate cancer, but the evidence is inconsistent. Our results, showing increased levels of many different fatty acids, along with higher amounts of plasma glycerol, emphasize the importance of altered lipid metabolism in the etiology of aggressive tumors.

The results of this study suggest a higher abundance of glycerol and glyceric acid, a glycerol oxidation product, in the plasma samples of patients diagnosed with prostate cancer, compared to healthy controls. Glyceric acid was found to be less abundant in metastatic breast cancer [58] and in the blood samples of individuals with advanced pancreatic cancer [59], which is not in line with our results that suggest higher levels of glyceric acid in patients with prostate cancer. Since we found the other carbohydrates and carbohydrate conjugates increased, one may assume that is a metabolic feature of prostate cancer presence. However, Huang and colleagues reported inverse associations between lethal prostate cancer risk and serum glycerol levels [60], so we should mention that all our patients survived the five years after surgery, which could be due to the proper therapy applied but also due to the less lethal character of the prostate cancer of our patients.

Furthermore, the level of different monoacylglycerols was found to be elevated in prostate cancer plasma samples. There is a lot of evidence supporting the role of monoacylglycerol lipase (MAGL) in tumorigenesis and metastasis. This lipolytic enzyme catalyzes the conversion of monoacylglycerides to free fatty acids and glycerol. The increased expression of MAGL was reported in diverse types of cancer, including prostate cancer [61]. Different fatty acids, glycerol and monoacylglycerols, which were found to be abundant in the prostate cancer plasma samples, could have a role as signaling molecules regulating cancer cell proliferation and aggressiveness.

A study by Crow and colleagues found a positive association between palmitic acid and prostate cancer risk and a positive association between α-linolenic acid and EPA with the risk of high-grade prostate cancer [62]. Non-targeted metabolomics analysis of the serum samples revealed 18 lipid or lipid-like compounds as potential biomarkers in the early diagnosis of prostate cancer [22]. These metabolites also included palmitic acid, which was found to be decreased in the prostate cancer samples, contrary to our results [22]. However, the above-mentioned study focused on metabolites that effectively discriminated prostate cancer patients from benign prostatic hyperplasia individuals. In the case of stearic acid, the lower proportion of this fatty acid was associated with a greater overall risk of prostate cancer [62], which is inconsistent with our results, indicating higher levels of this fatty acid in prostate cancer patients compared to healthy controls. The evidence regarding the association between dietary EPA and prostate cancer risk is inconclusive. Some studies reported a protective effect of EPA intake on prostate cancer [63,64,65]; however, others suggest a positive association between a higher intake of EPA and an increased risk of prostate cancer [66,67]. Circulating EPA levels have been positively associated with prostate cancer risk in a pooled analysis of individual data from prospective studies [68], and the dietary intake of EPA was associated with the risk of advanced or fatal prostate cancer in the NIH-American Association of Retired Persons (AARP) Diet and Health Study [69]. Elevated levels of 9-HODE and 13-HODE have been associated with increased prostate cancer mortality [70]. Interestingly, both 9-HODE and 13-HODE are synthesized from linoleic acid [71,72], a precursor of 4-HNE. The results showing a positive association of 9-HODE and 13-HODE serum concentration with increased risk for ovarian cancer are aligned with our results and highlight the importance of linoleic acid metabolites in the etiology of cancer [73].

A higher serum proportion of palmitoleic acid was associated with cancer mortality in the Swedish population [74], while oleic acid was found to promote the prostate cancer malignant phenotype of PC3 and DU-145 cells [75]. The intake of different short-chain fatty acids was shown to worsen prostate cancer survival [65], and higher intake of butyric acid, caproic acid, and the combined intake of different short-chain fatty acids (4:0–10:0) were associated with a higher risk of advanced prostate cancer [66].

We detected elevated levels of both 2-hydroxybutyric and 3-hydroxybutyric acid in patients diagnosed with prostate cancer. The 2-hydroxybutyric acid or α-hydroxybutyrate, which is produced by amino acid catabolism, was found to be elevated in the sera of colorectal cancer patients [76], and was suggested as a valuable biomarker for the early detection of colorectal cancer [77], as well as a potential early marker of insulin resistance and impaired glucose regulation, and was associated with elevated lipid oxidation and oxidative stress [78]. A meta-analysis of clinical metabolic profiling studies in 18 different cancer types, assessing blood, tissue, and urine samples, revealed that ketone bodies, which are produced during fatty acid metabolism, are metabolites most often found to be elevated in cancer compared to normal controls [79]. A ketone body 3-hydroxybutyric acid or β-hydroxybutyrate, which has been associated with tumor growth and cancer cachexia [80], was found to be upregulated in the blood samples from different cancer patients [79], and the same trend was detected in our study with patients diagnosed with prostate cancer.

Tumor metabolism is characterized by anaerobic glycolysis since cancer cells preferentially convert the glycolysis-induced pyruvate into lactic acid even in the presence of oxygen [59], resulting in elevated pericellular accumulation of organic acids, such as lactic acid, and low pyruvate status [81]. Our results also suggest a lower level of pyruvic acid in prostate cancer plasma samples if compared to healthy controls; however, contrary to our expectations, in our study, the levels of lactic acid were 56% lower in prostate cancer patients than in the healthy controls.

The blood level of glucose/galactose was elevated in prostate cancer patients compared with control subjects, confirming the positive associations between glucose and prostate cancer [82,83,84]. However, we also detected elevated levels of another hexose, mannose. Mannose, which is an isomer of glucose, was reported to have a potential anticancer effect on different tumors. Deng and colleagues demonstrated that mannose has an inhibitory effect on the proliferation and an apoptosis-promoting effect on prostate cancer cells in vitro [85]. The same trend was observed in mice [85]. However, high-throughput profiling of N-glycans in prostate cancer tissue samples revealed that high mannose glycans were more abundant in tumor regions than in non-tumor regions [86].

There has been a lot of evidence in the past decade supporting the role of cholesterol in prostate cancer and its progression [87,88,89,90,91,92]. Our results support the association between increased levels of blood cholesterol and prostate cancer diagnosis. Hypercholesterolemia could affect cancer cell proliferation [93,94], inflammation [95], lipid raft dynamics [96], and intertumoral steroidogenesis [97,98]. Different steroid hormones have a key role in the maintenance and progression of prostate cancer, and their effects are achieved through the androgen receptor [99]. The first step in steroidogenesis is the generation of the common steroid precursor pregnenolone from cholesterol. This common precursor of many steroids was found to induce the growth of LNCaP cells by binding to the mutated androgen receptor [100]. In our study, both cholesterol and pregnenolone levels were found to be much higher in patients with prostate cancer than in control subjects.

Hyodeoxycholic acid was found to be less abundant in patients with prostate cancer. This naturally occurring secondary bile acid is generated from lithocholic acid in the small intestine by the gut microbiota. Hyodeoxycholic acid is a weak liver X receptor α agonist with the capacity to decrease plasma glucose levels, improve plasma lipoprotein profiles, and increase the efficiency of intestinal cholesterol absorption [101]. Bile acids were primarily considered to promote carcinogenesis, but recently, more and more evidence suggests that lower concentrations of bile acids have potential anticancer activity, with an emphasis on ursodeoxycholic acid [102,103].

Alterations in amino acid metabolism are one of the hallmarks of cancer malignancy [104]. The 2-Ketoispcaproic acid, also known as α-ketoisocaproic acid or ketoleucine, is an abnormal metabolite associated with the incomplete breakdown of branched-chain amino acids. Ketoleucine was found to be increased in our group of patients diagnosed with prostate cancer, which is in accordance with the results from Jajin and colleagues, who detected increased 2-ketoisocaproic acid, α-ketoglutarate, and glutamine, along with decreased leucine and aspartate in medullary thyroid cancer [105]. In the last decade, the role of arginine in inflammation, cell activation, and growth has attracted a lot of attention due to the involvement of these processes in tumorigenesis [106,107]. The metabolism of arginine and the activity of arginase, the enzyme which hydrolyzes arginine to ornithine and urea, were found to be increased in prostate cancer and involved in differentiation [108,109,110]. In line with these studies, we also detected elevated levels of arginine in the plasma samples from prostate cancer patients. Dipeptide treonyhistidine was also found to be more abundant in the plasma of cancer patients than in healthy control subjects. The physical and functional properties of these dipeptides are different from those of single amino acids; however, their function in the human organism is not yet fully understood. Since our monoclonal antibody used in ELISA and immunohistochemistry is highly specific for the histidine adducts of 4-HNE [111], it might be possible that 4-HNE was, alongside 4-HNE-albumin and 4-HNE-LDL conjugates, also present in the plasma as a 4-HNE-treonyhistidine adduct. However, since there was no correlation between 4-HNE-His adducts determined by the ELISA and treonyhistidine levels determined by metabolomic analyses, we can assume that this dipeptide was not playing a major role in the formation of the 4-HNE-His adducts detected in the plasma of healthy controls and patients with prostate cancer. Different dipeptides were found to be elevated in the serum samples of prostate cancer patients [60], and recently, they have also attracted a lot of attention as potential disease biomarkers [112]. With respect to the derivatives of organic acids, it should be mentioned that levels of two phenylsulfates, O-methoxycatechol-O-sulphate, and pyrocatechol sulfate, were decreased significantly for 60–80% in the plasma of prostate cancer patients, but the biomedical relevance of these findings is still unknown.

Our results revealed elevated levels of biliverdin in patients diagnosed with prostate cancer. Biliverdin is a product of heme degradation by the heme oxygenase (HO) family of enzymes, so the increased level of biliverdin in cancer patients could be related to the altered activity of these enzymes. The HO-1 isoform was found to be expressed in a wide variety of cancers, including prostate cancer [113], and implicated in different biological processes which facilitate tumor progression and metastasis. The HO-1 possesses a dual role. It can prevent DNA damage and carcinogenesis in normal cells; however, in the late phase of tumorigenesis, the overexpression of HO-1 induces the proliferation and invasiveness of cancer cells [114,115,116].

Retinal belongs to the retinoid family, and it is the oxidized form of retinol. Retinoids have been shown to inhibit cell growth by inducing apoptosis and cellular differentiation, thus representing a potential approach to tumor therapy. However, the evidence of retinoids enhancing tumor growth [117] suggests a complicated and multifaceted role of retinoids in cancer. Our results indicate elevated levels of retinal in prostate cancer, which is partially in line with the findings suggesting the association of higher serum retinol with an elevated risk of prostate cancer [118].

In conclusion, our study revealed that the unsaturated fatty acids biosynthesis pathway could be modulated by elevated 4-HNE plasma protein adducts. This is a strong argument in favor of the 4-HNE analyses in clinical studies, as was suggested already years ago [119]. However, whether this is a direct effect of 4-HNE or they are simply induced by the same underlying mechanisms remains to be elucidated. Further mechanistic studies are needed to understand the biological relevance of the observed changes, which could also reveal with certainty the 4-HNE levels in the blood that might discriminate healthy from ill people, especially those suffering from cancer.

## Figures and Tables

**Table 1:** Significantly altered metabolites, detected by GC-MS, between patients with prostate cancer and healthy control subjects.

Category	Compound	RT	%Δ	FC	log_2_FC	pBH	VIP
Fatty Acyls	Caprylic acid (octanoic acid)	9.79	41.43	1.41	0.50	<0.0001	1.17
	Caproic acid (hexanoic acid)	7.06	−68.94	0.31	−1.69	<0.0001	2.04
	Lauric acid (dodecanoic acid)	14.75	69.22	1.69	0.76	0.002	1.00
	Palmitic acid (hexadecanoic acid)	18.87	92.58	1.93	0.95	<0.0001	1.06
	Stearic acid (octadecanoic acid)	20.69	80.19	1.80	0.85	0.002	1.02
	Palmitoleic acid (hexadecenoic acid)	18.68	181.07	2.81	1.49	<0.0001	1.00
	Linoleic acid (octadecadienoic acid)	20.41	140.74	2.41	1.27	<0.0001	1.02
	Oleic acid (octadecenoic acid)	20.46	177.32	2.77	1.47	<0.0001	1.07
Organic acids and derivatives	Lactic acid (2-hydroxypropanoic acid)	6.85	−56.24	0.44	−1.19	<0.0001	3.96
	2-hydroxybutyric acid	7.79	87.56	1.88	0.91	<0.0001	1.09
	3-hydroxybutyric acid	8.28	257.75	3.58	1.84	<0.0001	1.58
	Pyruvic acid (2-oxopropanoic acid)	6.70	−65.78	0.34	−1.55	<0.0001	2.04
	2-ketoisocaproic acid (ketoleucine)	8.54	33.61	1.34	0.42	0.049	1.03
Carbohydrates and carbohydrate conjugates	Glycerol	9.87	69.00	1.69	0.76	<0.0001	1.08
	Glyceric acid	10.65	30.93	1.31	0.39	0.002	1.00
	Mannose	17.22	19.80	1.20	0.26	0.002	1.02
	Galactose/glucose	17.55	17.57	1.18	0.23	0.002	1.66
Sterol Lipids	Cholesterol	27.57	30.47	1.30	0.38	0.003	1.04

%Δ, percentage of change; FC, fold change; pBH, Benjamini–Hochberg adjusted *p*-value; RT, retention time; VIP, variable importance in the projection.

**Table 2:** Significantly altered metabolites, detected by LC-MS, between patients with prostate cancer and healthy control subjects.

Category	Compound	ESI Mode	*m*/*z*	RT	%Δ	FC	log_2_FC	pBH	VIP
Fatty Acyls	Thapsic acid (hexadecanedioic acid)	-	285.2072	16.30	138.00	2.38	1.25	<0.0001	1.47
	Methylhexadecenoic acid	+	269.2461	18.70	86.68	1.87	0.90	<0.0001	1.05
	Palmitoleic acid	-	253.2176	27.60	88.09	1.88	0.91	<0.0001	1.14
	Linolenic acid (octadecatrienoic acid)	-	277.2174	25.90	91.04	1.91	0.93	<0.0001	1.12
	Oleic acid	-	281.2493	31.45	80.58	1.81	0.85	<0.0001	1.03
	Eicosapentaenoic acid	+	303.2313	25.54	252.02	3.52	1.82	<0.0001	1.19
	Docosapentaenoic acid	-	329.2488	28.63	103.96	2.04	1.03	<0.0001	1.19
	Decadienal	+	153.1267	12.45	−30.21	0.70	−0.52	<0.0001	0.28
	Octadecadienal (9,12)	+	265.2508	25.92	146.52	2.47	1.30	<0.0001	2.78
	Tetradecenoylcarnitine	+	370.2963	12.70	113.97	2.14	1.10	<0.0001	1.01
	9-hydroxyoctadecadienoic acid (9-HODE)	-	295.2278	18.71	388.27	4.88	2.29	<0.0001	3.97
Glycerolipids	MG(16:0)	+	331.2855	27.69	246.30	3.46	1.79	<0.0001	1.55
	MG(18:0)	+	359.3155	32.05	2966.39	30.66	4.94	<0.0001	6.00
	MG(18:2(9,12))	+	355.2844	25.33	238.69	3.39	1.76	<0.0001	1.23
Organic acids and derivatives	Arginine	+	175.1190	0.57	62.43	1.62	0.70	<0.0001	1.37
	Threonylhistidine	-	255.1121	1.79	91.05	1.91	0.93	<0.0001	1.57
	O-methoxycatechol-O-sulphate	-	203.0025	1.51	−71.64	0.28	−1.82	<0.0001	1.59
	Pyrocatechol sulfate	-	188.9878	1.19	−80.33	0.20	−2.35	<0.0001	3.47
Organoheterocyclic compounds	Biliverdin	+	583.2551	9.90	180.27	2.80	1.49	<0.0001	1.10
Prenol Lipids	Retinal	+	285.2222	25.55	754.02	8.54	3.09	<0.0001	2.91
Sterol Lipids	Hyodeoxycholic acid	-	391.2841	15.03	−83.22	0.17	−2.57	<0.0001	3.60
	Pregnenolone	+	317.2472	25.56	653.97	7.54	2.91	<0.0001	1.66

%Δ, percentage of change; FC, fold change; pBH, Benjamini–Hochberg adjusted *p*-value; RT, retention time; VIP, variable importance in the projection.

**Table 3:** Correlation between altered metabolites and levels of plasma 4-HNE-protein adducts.

	Healthy Controls			Prostate Cancer Patients		
Compound	r	95% Confidence Interval	*p*	r	95% Confidence Interval	*p*
2-ketoisocaproic acid	0.146	−0.179 to 0.442	0.363	0.394	0.005 to 0.680	0.042 *
9-HODE	−0.014	−0.376 to 0.352	0.941	0.633	0.343 to 0.812	0.000 ***
Caproic acid (hexanoic acid)	−0.312	−0.571 to 0.005	0.047 *	0.179	−0.227 to 0.532	0.371
Eicosapentaenoic acid	0.120	−0.208 to 0.424	0.460	0.544	0.211 to 0.764	0.002 **
Hexadecanedioic acid	0.169	−0.160 to 0.464	0.297	−0.421	−0.684 to −0.060	0.021 *
Lactic acid (2-hydroxypropanoic acid)	−0.149	−0.444 to 0.176	0.353	0.316	−0.085 to 0.628	0.109
Linoleic acid (octadecadienoic acid)	0.357	0.042 to 0.608	0.024 *	−0.115	−0.484 to 0.288	0.567
Methyl hexadecanoic acid	0.127	−0.312 to 0.522	0.562	0.597	0.261 to 0.804	0.001 **
MG(18:2(9,12))	0.124	−0.204 to 0.427	0.446	0.554	0.231 to 0.767	0.002 **
Octadecadienal (9,12)	0.189	−0.244 to 0.559	0.378	0.523	0.175 to 0.755	0.004 **
Octadecenoic acid	0.150	−0.174 to 0.445	0.349	−0.330	−0.623 to 0.046	0.075
Palmitoleic acid (hexadecenoic acid)	0.198	−0.144 to 0.498	0.239	−0.509	−0.750 to −0.148	0.007 **
Pregnenolone	0.280	−0.044 to 0.551	0.080	0.629	0.330 to 0.813	0.000 ***
Pyruvic acid (2-oxopropanoic acid)	−0.393	−0.630 to −0.087	0.011 *	−0.183	−0.535 to 0.223	0.361
Retinal	0.333	−0.057 to 0.635	0.083	0.553	0.207 to 0.776	0.003 **
Stearic acid (octadecanoic acid)	0.466	0.168 to 0.687	0.003 **	−0.106	−0.476 to 0.297	0.601

r, Spearman correlation coefficient; Significance: * *p* < 0.050; ** *p* < 0.010; *** *p* < 0.001.

## Data Availability

Not applicable.

## Supplementary Materials

The following supporting information can be downloaded at: https://www.mdpi.com/article/10.3390/biom13010145/s1, Table S1: General characteristics of prostate cancer patients and immunohistochemical appearance of 4-HNE in cancer tissue samples, Figure S1: Validity tests for OPLS-DA models. Plots were obtained using SIMCA-P+ software (version 15.0.2.5959, Umetrics, Umea, Sweden).

## References

[1] Sung H., Ferlay J., Siegel R.L., Laversanne M., Soerjomataram I., Jemal A., Bray F. (2021). Global Cancer Statistics 2020: GLOBOCAN Estimates of Incidence and Mortality Worldwide for 36 Cancers in 185 Countries. CA Cancer J. Clin..

[2] Rawla P. (2019). Epidemiology of Prostate Cancer. World J. Oncol..

[3] Netto G., Amin M., Kench J., Al E., Srigley J., Amin M., Rubin M., Tsuzuki T. (2022). Tumours of the prostate. WHO Classification of Tumours: Urinary and Male Genital Tumours.

[4] Ström P., Kartasalo K., Olsson H., Solorzano L., Delahunt B., Berney D.M., Bostwick D.G., Evans A.J., Grignon D.J., Humphrey P.A. (2020). Artificial intelligence for diagnosis and grading of prostate cancer in biopsies: A population-based, diagnostic study. Lancet. Oncol..

[5] Jaganjac M., Poljak-Blazi M., Zarkovic K., Schaur R.J., Zarkovic N. (2008). The involvement of granulocytes in spontaneous regression of Walker 256 carcinoma. Cancer Lett..

[6] Žarković N., Jaganjac M., Žarković K., Gęgotek A., Skrzydlewska E. (2022). Spontaneous Regression of Cancer: Revealing Granulocytes and Oxidative Stress as the Crucial Double-edge Sword. Front. Biosci..

[7] Costanzo-Garvey D.L., Case A.J., Watson G.F., Alsamraae M., Chatterjee A., Oberley-Deegan R.E., Dutta S., Abdalla M.Y., Kielian T., Lindsey M.L. (2022). Prostate cancer addiction to oxidative stress defines sensitivity to anti-tumor neutrophils. Clin. Exp. Metastasis.

[8] Jaganjac M., Cindrić M., Jakovčević A., Žarković K., Žarković N. (2021). Lipid peroxidation in brain tumors. Neurochem. Int..

[9] Hacer İ.A., Zeynep A.A., Can Ö., Riza K.A., Dildar K., Tülay A. (2003). The effect of prostate cancer and antianrogenic therapy on lipid peroxidation and antioxidant systems. Int. Urol. Nephrol..

[10] Srivastava D.S.L., Mittal R.D. (2005). Free radical injury and antioxidant status in patients with benign prostate hyperplasia and prostate cancer. Indian J. Clin. Biochem..

[11] Custovic Z., Zarkovic K., Cindric M., Cipak A., Jurkovic I., Sonicki Z., Uchida K., Zarkovic N. (2010). Lipid peroxidation product acrolein as a predictive biomarker of prostate carcinoma relapse after radical surgery. Free Radic. Res..

[12] Jaganjac M., Milkovic L., Gegotek A., Cindric M., Zarkovic K., Skrzydlewska E., Zarkovic N. (2020). The relevance of pathophysiological alterations in redox signaling of 4-hydroxynonenal for pharmacological therapies of major stress-associated diseases. Free Radic. Biol. Med..

[13] Zarkovic K., Jakovcevic A., Zarkovic N. (2017). Contribution of the HNE-immunohistochemistry to modern pathological concepts of major human diseases. Free Radic. Biol. Med..

[14] Schaur R.J., Siems W., Bresgen N., Eckl P.M. (2015). 4-Hydroxy-nonenal-A Bioactive Lipid Peroxidation Product. Biomolecules.

[15] Peiro G., Alary J., Cravedi J.-P., Rathahao E., Steghens J.-P., Guéraud F. (2005). Dihydroxynonene mercapturic acid, a urinary metabolite of 4-hydroxynonenal, as a biomarker of lipid peroxidation. Biofactors.

[16] Pierre F., Peiro G., Taché S., Cross A.J., Bingham S.A., Gasc N., Gottardi G., Corpet D.E., Guéraud F. (2006). New marker of colon cancer risk associated with heme intake: 1,4-dihydroxynonane mercapturic acid. Cancer Epidemiol. Biomark. Prev..

[17] Cherkas A., Golota S., Guéraud F., Abrahamovych O., Pichler C., Nersesyan A., Krupak V., Bugiichyk V., Yatskevych O., Pliatsko M. (2018). A Helicobacter pylori-associated insulin resistance in asymptomatic sedentary young men does not correlate with inflammatory markers and urine levels of 8-iso-PGF(2)-α or 1,4-dihydroxynonane mercapturic acid. Arch. Physiol. Biochem..

[18] Al-Menhali A.S., Anderson C., Gourine A.V., Abramov A.Y., D’Souza A., Jaganjac M. (2021). Proteomic Analysis of Cardiac Adaptation to Exercise by High Resolution Mass Spectrometry. Front. Mol. Biosci..

[19] Gęgotek A., Domingues P., Wroński A., Ambrożewicz E., Skrzydlewska E. (2019). The Proteomic Profile of Keratinocytes and Lymphocytes in Psoriatic Patients. Proteom. Clin. Appl..

[20] Hauck A.K., Zhou T., Upadhyay A., Sun Y., O’connor M.B., Chen Y., Bernlohr D.A. (2020). Histone carbonylation is a redox-regulated epigenomic mark that accumulates with obesity and aging. Antioxidants.

[21] Wang Y., Jacobs E.J., Carter B.D., Gapstur S.M., Stevens V.L. (2021). Plasma Metabolomic Profiles and Risk of Advanced and Fatal Prostate Cancer. Eur. Urol. Oncol..

[22] Xu B., Chen Y., Chen X., Gan L., Zhang Y., Feng J., Yu L. (2021). Metabolomics Profiling Discriminates Prostate Cancer From Benign Prostatic Hyperplasia Within the Prostate-Specific Antigen Gray Zone. Front. Oncol..

[23] Walz S., Wang Q., Zhao X., Hoene M., Häring H.-U., Hennenlotter J., Maas M., Peter A., Todenhöfer T., Stenzl A. (2021). Comparison of the metabolome in urine prior and eight weeks after radical prostatectomy uncovers pathologic and molecular features of prostate cancer. J. Pharm. Biomed. Anal..

[24] Falegan O.S., Jarvi K., Vogel H.J., Hyndman M.E. (2021). Seminal plasma metabolomics reveals lysine and serine dysregulation as unique features distinguishing between prostate cancer tumors of Gleason grades 6 and 7. Prostate.

[25] Zarkovic K., Juric G., Waeg G., Kolenc D., Zarkovic N. (2005). Immunohistochemical appearance of HNE-protein conjugates in human astrocytomas. BioFactors.

[26] Weber D., Milkovic L., Bennett S.J., Griffiths H.R., Zarkovic N., Grune T. (2013). Measurement of HNE-protein adducts in human plasma and serum by ELISA-Comparison of two primary antibodies. Redox Biol..

[27] Perković M.N., Milković L., Uzun S., Mimica N., Pivac N., Waeg G., Žarković N. (2021). Association of Lipid Peroxidation Product 4-Hydroxynonenal with Post-Traumatic Stress Disorder. Biomolecules.

[28] Gil de la Fuente A., Godzien J., Fernández López M., Rupérez F.J., Barbas C., Otero A. (2018). Knowledge-based metabolite annotation tool: CEU Mass Mediator. J. Pharm. Biomed. Anal..

[29] Naz S., García A., Barbas C. (2013). Multiplatform analytical methodology for metabolic fingerprinting of lung tissue. Anal. Chem..

[30] Wishart D.S., Feunang Y.D., Marcu A., Guo A.C., Liang K., Vázquez-Fresno R., Sajed T., Johnson D., Li C., Karu N. (2018). HMDB 4.0: The human metabolome database for 2018. Nucleic Acids Res..

[31] Smith C.A., O’Maille G., Want E.J., Qin C., Trauger S.A., Brandon T.R., Custodio D.E., Abagyan R., Siuzdak G. (2005). METLIN: A metabolite mass spectral database. Ther. Drug Monit..

[32] Kanehisa M., Goto S. (2000). KEGG: Kyoto encyclopedia of genes and genomes. Nucleic Acids Res..

[33] Fahy E., Sud M., Cotter D., Subramaniam S. (2007). LIPID MAPS online tools for lipid research. Nucleic Acids Res..

[34] Godzien J., Alonso-Herranz V., Barbas C., Armitage E.G. (2015). Controlling the quality of metabolomics data: New strategies to get the best out of the QC sample. Metabolomics.

[35] Kuligowski J., Sánchez-Illana Á., Sanjuán-Herráez D., Vento M., Quintás G. (2015). Intra-batch effect correction in liquid chromatography-mass spectrometry using quality control samples and support vector regression (QC-SVRC). Analyst.

[36] Chang C.-C., Lin C.-J. (2011). LIBSVM: A library for support vector machines. ACM Trans. Intell. Syst. Technol..

[37] De Livera A.M., Dias D.A., De Souza D., Rupasinghe T., Pyke J., Tull D., Roessner U., McConville M., Speed T.P. (2012). Normalizing and integrating metabolomics data. Anal. Chem..

[38] Gromski P.S., Xu Y., Hollywood K.A., Turner M.L., Goodacre R. (2015). The influence of scaling metabolomics data on model classification accuracy. Metabolomics.

[39] Pang Z., Chong J., Zhou G., de Lima Morais D.A., Chang L., Barrette M., Gauthier C., Jacques P.-É., Li S., Xia J. (2021). MetaboAnalyst 5.0: Narrowing the gap between raw spectra and functional insights. Nucleic Acids Res..

[40] Pang Z., Zhou G., Ewald J., Chang L., Hacariz O., Basu N., Xia J. (2022). Using MetaboAnalyst 5.0 for LC–HRMS spectra processing, multi-omics integration and covariate adjustment of global metabolomics data. Nat. Protoc..

[41] Živković N.P., Petrovečki M., Lončarić Č.T., Nikolić I., Waeg G., Jaganjac M., Žarković K., Žarković N. (2017). Positron emission tomography-computed tomography and 4-hydroxynonenal-histidine immunohistochemistry reveal differential onset of lipid peroxidation in primary lung cancer and in pulmonary metastasis of remote malignancies. Redox Biol..

[42] Zhong H., Xiao M., Zarkovic K., Zhu M., Sa R., Lu J., Tao Y., Chen Q., Xia L., Cheng S. (2017). Mitochondrial control of apoptosis through modulation of cardiolipin oxidation in hepatocellular carcinoma: A novel link between oxidative stress and cancer. Free Radic. Biol. Med..

[43] Gęgotek A., Nikliński J., Žarković N., Žarković K., Waeg G., Łuczaj W., Charkiewicz R., Skrzydlewska E. (2016). Lipid mediators involved in the oxidative stress and antioxidant defence of human lung cancer cells. Redox Biol..

[44] Bellezza I., Scarpelli P., Pizzo S.V., Grottelli S., Costanzi E., Minelli A. (2017). ROS-independent Nrf2 activation in prostate cancer. Oncotarget.

[45] Pettazzoni P., Ciamporcero E., Medana C., Pizzimenti S., Dal Bello F., Minero V.G., Toaldo C., Minelli R., Uchida K., Dianzani M.U. (2011). Nuclear factor erythroid 2-related factor-2 activity controls 4-hydroxynonenal metabolism and activity in prostate cancer cells. Free Radic. Biol. Med..

[46] Sovic A., Borovic S., Loncaric I., Kreuzer T., Zarkovic K., Vukovic T., Wäg G., Hrascan R., Wintersteiger R., Klinger R. (2001). The carcinostatic and proapoptotic potential of 4-hydroxynonenal in HeLa cells is associated with its conjugation to cellular proteins. Anticancer Res..

[47] Sunjic S.B., Gasparovic A.C., Jaganjac M., Rechberger G., Meinitzer A., Grune T., Kohlwein S.D., Mihaljevic B., Zarkovic N. (2021). Sensitivity of Osteosarcoma Cells to Concentration-Dependent Bioactivities of Lipid Peroxidation Product 4-Hydroxynonenal Depend on Their Level of Differentiation. Cells.

[48] Sunjic S.B., Cipak A., Rabuzin F., Wildburger R., Zarkovic N. (2005). The influence of 4-hydroxy-2-nonenal on proliferation, differentiation and apoptosis of human osteosarcoma cells. Biofactors.

[49] Tirumalai R., Rajesh Kumar T., Mai K.H., Biswal S. (2002). Acrolein causes transcriptional induction of phase II genes by activation of Nrf2 in human lung type II epithelial (A549) cells. Toxicol. Lett..

[50] Zarkovic K., Uchida K., Kolenc D., Hlupic L., Zarkovic N. (2006). Tissue distribution of lipid peroxidation product acrolein in human colon carcinogenesis. Free Radic. Res..

[51] Tabor C.W., Tabor H., Bachrach U. (1964). Identification of the aminoaldehydes produced by the oxidation of spermine and spermidine with purified plasma amine oxidase. J. Biol. Chem..

[52] Jaganjac M., Poljak-Blazi M., Schaur R.J., Zarkovic K., Borovic S., Cipak A., Cindric M., Uchida K., Waeg G., Zarkovic N. (2012). Elevated neutrophil elastase and acrolein-protein adducts are associated with W256 regression. Clin. Exp. Immunol..

[53] Jaganjac M., Matijevic Glavan T., Zarkovic N. (2019). The Role of Acrolein and NADPH Oxidase in the Granulocyte-Mediated Growth-Inhibition of Tumor Cells. Cells.

[54] Bauer G., Zarkovic N. (2015). Revealing mechanisms of selective, concentration-dependent potentials of 4-hydroxy-2-nonenal to induce apoptosis in cancer cells through inactivation of membrane-associated catalase. Free Radic. Biol. Med..

[55] Jaganjac M., Almuraikhy S., Al-Khelaifi F., Al-Jaber M., Bashah M., Mazloum N.A., Zarkovic K., Zarkovic N., Waeg G., Kafienah W. (2017). Combined metformin and insulin treatment reverses metabolically impaired omental adipogenesis and accumulation of 4-hydroxynonenal in obese diabetic patients. Redox Biol..

[56] Zarkovic N., Jakovcevic A., Mataic A., Jaganjac M., Vukovic T., Waeg G., Zarkovic K. (2022). Post-mortem Findings of Inflammatory Cells and the Association of 4-Hydroxynonenal with Systemic Vascular and Oxidative Stress in Lethal COVID-19. Cells.

[57] Žarković N., Orehovec B., Baršić B., Tarle M., Kmet M., Lukšić I., Tatzber F., Wonisch W., Skrzydlewska E., Łuczaj W. (2022). Lipidomics Revealed Plasma Phospholipid Profile Differences between Deceased and Recovered COVID-19 Patients. Biomolecules.

[58] Kim H.-Y., Lee K.-M., Kim S.-H., Kwon Y.-J., Chun Y.-J., Choi H.-K. (2016). Comparative metabolic and lipidomic profiling of human breast cancer cells with different metastatic potentials. Oncotarget.

[59] Ikeda A., Nishiumi S., Shinohara M., Yoshie T., Hatano N., Okuno T., Bamba T., Fukusaki E., Takenawa T., Azuma T. (2012). Serum metabolomics as a novel diagnostic approach for gastrointestinal cancer. Biomed. Chromatogr..

[60] Huang J., Mondul A.M., Weinstein S.J., Derkach A., Moore S.C., Sampson J.N., Albanes D. (2019). Prospective serum metabolomic profiling of lethal prostate cancer. Int. J. Cancer.

[61] Nomura D.K., Lombardi D.P., Chang J.W., Niessen S., Ward A.M., Long J.Z., Hoover H.H., Cravatt B.F. (2011). Monoacylglycerol Lipase Exerts Dual Control over Endocannabinoid and Fatty Acid Pathways to Support Prostate Cancer. Chem. Biol..

[62] Crowe F.L., Allen N.E., Appleby P.N., Overvad K., Aardestrup I.V., Johnsen N.F., Tjønneland A., Linseisen J., Kaaks R., Boeing H. (2008). Fatty acid composition of plasma phospholipids and risk of prostate cancer in a case-control analysis nested within the European Prospective Investigation into Cancer and Nutrition. Am. J. Clin. Nutr..

[63] Leitzmann M.F., Stampfer M.J., Michaud D.S., Augustsson K., Colditz G.C., Willett W.C., Giovannucci E.L. (2004). Dietary intake of n-3 and n-6 fatty acids and the risk of prostate cancer. Am. J. Clin. Nutr..

[64] Augustsson K., Michaud D.S., Rimm E.B., Leitzmann M.F., Stampfer M.J., Willett W.C., Giovannucci E. (2003). A prospective study of intake of fish and marine fatty acids and prostate cancer. Cancer Epidemiol. Biomark. Prev..

[65] Epstein M.M., Kasperzyk J.L., Mucci L.A., Giovannucci E., Price A., Wolk A., Håkansson N., Fall K., Andersson S.-O., Andrén O. (2012). Dietary fatty acid intake and prostate cancer survival in Örebro County, Sweden. Am. J. Epidemiol..

[66] Perez-Cornago A., Huybrechts I., Appleby P.N., Schmidt J.A., Crowe F.L., Overvad K., Tjønneland A., Kühn T., Katzke V., Trichopoulou A. (2020). Intake of individual fatty acids and risk of prostate cancer in the European prospective investigation into cancer and nutrition. Int. J. Cancer.

[67] Wallström P., Bjartell A., Gullberg B., Olsson H., Wirfält E. (2007). A prospective study on dietary fat and incidence of prostate cancer (Malmö, Sweden). Cancer Causes Control.

[68] Crowe F.L., Appleby P.N., Travis R.C., Barnett M., Brasky T.M., Bueno-de-Mesquita H.B., Chajes V., Chavarro J.E., Chirlaque M.-D., English D.R. (2014). Circulating fatty acids and prostate cancer risk: Individual participant meta-analysis of prospective studies. J. Natl. Cancer Inst..

[69] Pelser C., Mondul A.M., Hollenbeck A.R., Park Y. (2013). Dietary fat, fatty acids, and risk of prostate cancer in the NIH-AARP diet and health study. Cancer Epidemiol. Biomark. Prev..

[70] Huang J., Zhao B., Weinstein S.J., Albanes D., Mondul A.M. (2022). Metabolomic profile of prostate cancer-specific survival among 1812 Finnish men. BMC Med..

[71] Kelavkar U.P., Nixon J.B., Cohen C., Dillehay D., Eling T.E., Badr K.F. (2001). Overexpression of 15-lipoxygenase-1 in PC-3 human prostate cancer cells increases tumorigenesis. Carcinogenesis.

[72] Kelavkar U.P., Hutzley J., McHugh K., Allen K.G.D., Parwani A. (2009). Prostate tumor growth can be modulated by dietarily targeting the 15-lipoxygenase-1 and cyclooxygenase-2 enzymes. Neoplasia.

[73] Hada M., Edin M.L., Hartge P., Lih F.B., Wentzensen N., Zeldin D.C., Trabert B. (2019). Prediagnostic Serum Levels of Fatty Acid Metabolites and Risk of Ovarian Cancer in the Prostate, Lung, Colorectal, and Ovarian (PLCO) Cancer Screening Trial. Cancer Epidemiol. Biomark. Prev..

[74] Byberg L., Kilander L., Warensjö Lemming E., Michaëlsson K., Vessby B. (2014). Cancer death is related to high palmitoleic acid in serum and to polymorphisms in the SCD-1 gene in healthy Swedish men. Am. J. Clin. Nutr..

[75] Liotti A., Cosimato V., Mirra P., Calì G., Conza D., Secondo A., Luongo G., Terracciano D., Formisano P., Beguinot F. (2018). Oleic acid promotes prostate cancer malignant phenotype via the G protein-coupled receptor FFA1/GPR40. J. Cell. Physiol..

[76] Qiu Y., Cai G., Su M., Chen T., Zheng X., Xu Y., Ni Y., Zhao A., Xu L.X., Cai S. (2009). Serum metabolite profiling of human colorectal cancer using GC-TOFMS and UPLC-QTOFMS. J. Proteome Res..

[77] Nishiumi S., Kobayashi T., Ikeda A., Yoshie T., Kibi M., Izumi Y., Okuno T., Hayashi N., Kawano S., Takenawa T. (2012). A novel serum metabolomics-based diagnostic approach for colorectal cancer. PLoS ONE.

[78] Gall W.E., Beebe K., Lawton K.A., Adam K.-P., Mitchell M.W., Nakhle P.J., Ryals J.A., Milburn M.V., Nannipieri M., Camastra S. (2010). Alpha-hydroxybutyrate is an early biomarker of insulin resistance and glucose intolerance in a nondiabetic population. PLoS ONE.

[79] Goveia J., Pircher A., Conradi L.-C., Kalucka J., Lagani V., Dewerchin M., Eelen G., DeBerardinis R.J., Wilson I.D., Carmeliet P. (2016). Meta-analysis of clinical metabolic profiling studies in cancer: Challenges and opportunities. EMBO Mol. Med..

[80] Shukla S.K., Gebregiworgis T., Purohit V., Chaika N.V., Gunda V., Radhakrishnan P., Mehla K., Pipinos I.I., Powers R., Yu F. (2014). Metabolic reprogramming induced by ketone bodies diminishes pancreatic cancer cachexia. Cancer Metab..

[81] Schornack P.A., Gillies R.J. (2003). Contributions of cell metabolism and H+ diffusion to the acidic pH of tumors. Neoplasia.

[82] Bensimon L., Yin H., Suissa S., Pollak M.N., Azoulay L. (2014). Type 2 diabetes and the risk of mortality among patients with prostate cancer. Cancer Causes Control.

[83] Cai H., Xu Z., Xu T., Yu B., Zou Q. (2015). Diabetes mellitus is associated with elevated risk of mortality amongst patients with prostate cancer: A meta-analysis of 11 cohort studies. Diabetes. Metab. Res. Rev..

[84] Arthur R., Møller H., Garmo H., Häggström C., Holmberg L., Stattin P., Malmström H., Lambe M., Hammar N., Walldius G. (2019). Serum glucose, triglycerides, and cholesterol in relation to prostate cancer death in the Swedish AMORIS study. Cancer Causes Control.

[85] Deng Y.-L., Liu R., Cai Z.-D., Han Z.-D., Feng Y.-F., Cai S.-H., Chen Q.-B., Zhu J.-G., Zhong W.-D. (2022). Mannose inhibits the growth of prostate cancer through a mitochondrial mechanism. Asian J. Androl..

[86] Conroy L.R., Stanback A.E., Young L.E.A., Clarke H.A., Austin G.L., Liu J., Allison D.B., Sun R.C. (2021). In Situ Analysis of N-Linked Glycans as Potential Biomarkers of Clinical Course in Human Prostate Cancer. Mol. Cancer Res..

[87] Platz E.A., Till C., Goodman P.J., Parnes H.L., Figg W.D., Albanes D., Neuhouser M.L., Klein E.A., Thompson I.M.J., Kristal A.R. (2009). Men with low serum cholesterol have a lower risk of high-grade prostate cancer in the placebo arm of the prostate cancer prevention trial. Cancer Epidemiol. Biomark. Prev..

[88] Mondul A.M., Clipp S.L., Helzlsouer K.J., Platz E.A. (2010). Association between plasma total cholesterol concentration and incident prostate cancer in the CLUE II cohort. Cancer Causes Control.

[89] Van Hemelrijck M., Walldius G., Jungner I., Hammar N., Garmo H., Binda E., Hayday A., Lambe M., Holmberg L. (2011). Low levels of apolipoprotein A-I and HDL are associated with risk of prostate cancer in the Swedish AMORIS study. Cancer Causes Control.

[90] Batty G.D., Kivimäki M., Clarke R., Davey Smith G., Shipley M.J. (2011). Modifiable risk factors for prostate cancer mortality in London: Forty years of follow-up in the Whitehall study. Cancer Causes Control.

[91] Farwell W.R., D’Avolio L.W., Scranton R.E., Lawler E.V., Gaziano J.M. (2011). Statins and prostate cancer diagnosis and grade in a veterans population. J. Natl. Cancer Inst..

[92] Shafique K., McLoone P., Qureshi K., Leung H., Hart C., Morrison D.S. (2012). Cholesterol and the risk of grade-specific prostate cancer incidence: Evidence from two large prospective cohort studies with up to 37 years’ follow up. BMC Cancer.

[93] Solomon K.R., Freeman M.R. (2011). The complex interplay between cholesterol and prostate malignancy. Urol. Clin. North Am..

[94] Pelton K., Freeman M.R., Solomon K.R. (2012). Cholesterol and prostate cancer. Curr. Opin. Pharmacol..

[95] Freeman M.R., Solomon K.R. (2011). Cholesterol and benign prostate disease. Differentiation.

[96] Zhuang L., Lin J., Lu M.L., Solomon K.R., Freeman M.R. (2002). Cholesterol-rich lipid rafts mediate akt-regulated survival in prostate cancer cells. Cancer Res..

[97] Locke J.A., Guns E.S., Lubik A.A., Adomat H.H., Hendy S.C., Wood C.A., Ettinger S.L., Gleave M.E., Nelson C.C. (2008). Androgen levels increase by intratumoral de novo steroidogenesis during progression of castration-resistant prostate cancer. Cancer Res..

[98] Montgomery R.B., Mostaghel E.A., Vessella R., Hess D.L., Kalhorn T.F., Higano C.S., True L.D., Nelson P.S. (2008). Maintenance of intratumoral androgens in metastatic prostate cancer: A mechanism for castration-resistant tumor growth. Cancer Res..

[99] Snaterse G., Visser J.A., Arlt W., Hofland J. (2017). Circulating steroid hormone variations throughout different stages of prostate cancer. Endocr. Relat. Cancer.

[100] Grigoryev D.N., Long B.J., Njar V.C.O., Brodie A.H.M. (2000). Pregnenolone stimulates LNCaP prostate cancer cell growth via the mutated androgen receptor. J. Steroid Biochem. Mol. Biol..

[101] Shih D.M., Shaposhnik Z., Meng Y., Rosales M., Wang X., Wu J., Ratiner B., Zadini F., Zadini G., Lusis A.J. (2013). Hyodeoxycholic acid improves HDL function and inhibits atherosclerotic lesion formation in LDLR-knockout mice. FASEB J. Off. Publ. Fed. Am. Soc. Exp. Biol..

[102] Režen T., Rozman D., Kovács T., Kovács P., Sipos A., Bai P., Mikó E. (2022). The role of bile acids in carcinogenesis. Cell. Mol. Life Sci..

[103] Fu J., Yu M., Xu W., Yu S. (2021). Research Progress of Bile Acids in Cancer. Front. Oncol..

[104] Wei Z., Liu X., Cheng C., Yu W., Yi P. (2020). Metabolism of Amino Acids in Cancer. Front. Cell Dev. Biol..

[105] Jajin M.G., Abooshahab R., Hooshmand K., Moradi A., Siadat S.D., Mirzazadeh R., Chegini K.G., Hedayati M. (2022). Gas chromatography-mass spectrometry-based untargeted metabolomics reveals metabolic perturbations in medullary thyroid carcinoma. Sci. Rep..

[106] Capuano G., Rigamonti N., Grioni M., Freschi M., Bellone M. (2009). Modulators of arginine metabolism support cancer immunosurveillance. BMC Immunol..

[107] Wang B., Rong X., Palladino E.N.D., Wang J., Fogelman A.M., Martín M.G., Alrefai W.A., Ford D.A., Tontonoz P. (2018). Phospholipid Remodeling and Cholesterol Availability Regulate Intestinal Stemness and Tumorigenesis. Cell Stem Cell.

[108] Bronte V., Kasic T., Gri G., Gallana K., Borsellino G., Marigo I., Battistini L., Iafrate M., Prayer-Galetti T., Pagano F. (2005). Boosting antitumor responses of T lymphocytes infiltrating human prostate cancers. J. Exp. Med..

[109] Mumenthaler S.M., Yu H., Tze S., Cederbaum S.D., Pegg A.E., Seligson D.B., Grody W.W. (2008). Expression of arginase II in prostate cancer. Int. J. Oncol..

[110] Gannon P.O., Godin-Ethier J., Hassler M., Delvoye N., Aversa M., Poisson A.O., Péant B., Alam Fahmy M., Saad F., Lapointe R. (2010). Androgen-regulated expression of arginase 1, arginase 2 and interleukin-8 in human prostate cancer. PLoS ONE.

[111] Waeg G., Dimsity G., Esterbauer H. (1996). Monoclonal antibodies for detection of 4-hydroxynonenal modified proteins. Free Radic. Res..

[112] Tang Y., Li R., Lin G., Li L. (2014). PEP search in MyCompoundID: Detection and identification of dipeptides and tripeptides using dimethyl labeling and hydrophilic interaction liquid chromatography tandem mass spectrometry. Anal. Chem..

[113] Wegiel B., Gallo D., Csizmadia E., Harris C., Belcher J., Vercellotti G.M., Penacho N., Seth P., Sukhatme V., Ahmed A. (2013). Carbon monoxide expedites metabolic exhaustion to inhibit tumor growth. Cancer Res..

[114] Sunamura M., Duda D.G., Ghattas M.H., Lozonschi L., Motoi F., Yamauchi J.-I., Matsuno S., Shibahara S., Abraham N.G. (2003). Heme oxygenase-1 accelerates tumor angiogenesis of human pancreatic cancer. Angiogenesis.

[115] Chen G.G., Liu Z.M., Vlantis A.C., Tse G.M.K., Leung B.C.H., van Hasselt C.A. (2004). Heme oxygenase-1 protects against apoptosis induced by tumor necrosis factor-alpha and cycloheximide in papillary thyroid carcinoma cells. J. Cell. Biochem..

[116] Was H., Cichon T., Smolarczyk R., Rudnicka D., Stopa M., Chevalier C., Leger J.J., Lackowska B., Grochot A., Bojkowska K. (2006). Overexpression of heme oxygenase-1 in murine melanoma: Increased proliferation and viability of tumor cells, decreased survival of mice. Am. J. Pathol..

[117] Matsumoto T., Mochizuki W., Nibe Y., Akiyama S., Matsumoto Y., Nozaki K., Fukuda M., Hayashi A., Mizutani T., Oshima S. (2016). Retinol Promotes In Vitro Growth of Proximal Colon Organoids through a Retinoic Acid-Independent Mechanism. PLoS ONE.

[118] Mondul A.M., Watters J.L., Männistö S., Weinstein S.J., Snyder K., Virtamo J., Albanes D. (2011). Serum retinol and risk of prostate cancer. Am. J. Epidemiol..

[119] Frijhoff J., Winyard P.G., Zarkovic N., Davies S., Stocker R., Cheng D., Knight A., Taylor E.L., Oettrich J., Ruskovska T. (2015). Clinical relevance of biomarkers of oxidative stress. Antioxid. Redox Signal..

