# Supplementary material for: Relationship between 4-Hydroxynonenal (4-HNE) as Systemic Biomarker of Lipid Peroxidation and Metabolomic Profiling of Patients with Prostate Cancer
Source: Biomolecules. 2023 Jan 10;13(1):145. doi: 10.3390/biom13010145 (PMC9855859; doi:10.3390/biom13010145)
Supplement: Supplementary file 1 [file biomolecules-13-00145-s001.zip › biomolecules-2100381-supplementary.pdf]

biomolecules

MDPI

Supplementary materials

Table S1. Clinical features of the patients with prostate cancer involved in the study and 4-HNE immunohistochemistry results obtained.

Patient number	Age	PSA*	Cancer stage**	4-HNE in cancer cells***	4-HNE in stroma	4-HNE in blood vessels
1	63	5.82	GS 3+4=7(pT2c+N0MX).	0	1	1
2	64	5.22	GS 3+4=7(pT2c N0MX).	0	0	0
3	57	6.3	GS 3+4=7(pT3aN0MxR+).	0	0	0
4	64	4.96	GS 3+3=6(pT2cNoMX R+).	0	0	0
5	57	7.4	GS 4+3=7(pT2a NoMX).	0	0	0
6	62	12	GS 3+4=7pT(m)2cN0MXR+	0	0	0
7	59	4.03	GS 4+3=7pT2cN0Mx	0	0	0
8	62	41	GS 3+4=7pT2cN0MxR+	0	0	0
9	74	4.6	GS4+3=7 pT2aN0Mx	0	0	0
10	64	5.3	GS 3+4=7 (pT2c N0MX).	0	0	0
11	62	7	GS 3+4=7 (pT2c N0MX).	0	0	0
12	68	8.33	GS 3+4=7 (pT2c N0MX).	0	0	0
13	70	5.67	GS 3+4=7 (pT2c N0MX).	0	0	0
14	67	5.21	GS 3+4=7 (pT3aN0MxR1).	0	0	0
15	66	4.79	GS 3+4=7(pT2cN0MxR0).	0	0	0
16	69	25.12	GS 4+3=7 (pT2cN0Mx)	0	0	0
17	62	8.7	GS 3+4=7 pT3aN0MxR+)	0	0	0
18	61	9.78	GS 3+5=8 pT3bN1MxR+)	0	0	0
19	60	8.67	GS 3+4=7 pT2aN0Mx	0	0	0
20	61	8.97	GS 3+4=7 (pT2c N0MX).	0	0	0
21	61	5.4	GS 3+4=7 (pT2c N0MX).	0	0	0
22	63	5.79	GS 3+4=7 (pT2c N0MX).	0	0	0
23	68	4	GS 3+4=7 (pT2c N0MX).	0	0	0
24	64	12.7	GS 3+3=6 (pT2c N0MX).	0	0	0
25	64	7.08	GS 3+4=7 (pT2N0Mx)	0	0	0
26	67	8	GS 3+4=7 (pT2c N0MX).	0	0	0
27	64	4.25	GS 3+4=7 (pT2c N0MX).	0	0	0
28	50	3.5	GS 3+4=7 (pT2N0Mx)	0	0	0
29	56	8.6	GS 4+4=8 (pT3bN0Mx)	0	0	0
30	65	6.2	GS 3+4=7 (pT2c NxMX).	0	0	0

* PSA values determined for the blood samples collected before surgery (ng/mL)
** G=Gleason values of tumor differentiation summarizing values of two respective cancer areas, pTNM – as determined by pathohistology does not reflect possible remote metastases
*** Immunohistochemical findings of 1 means low incidence of HNE-immunopositivity, while 0 means no positivity at all

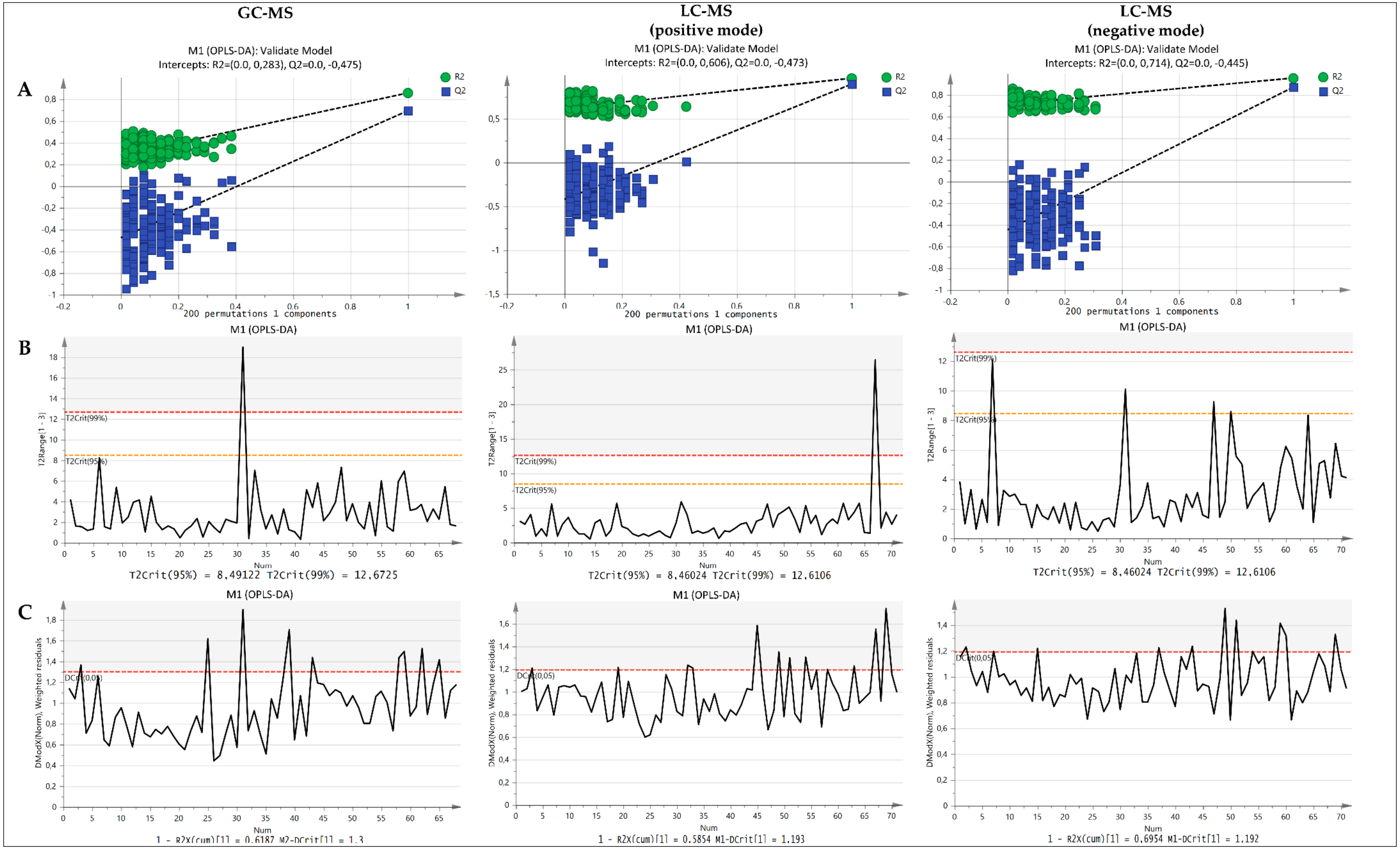

Figure S1. Validity tests for OPLS-DA models. Plots were obtained using SIMCA-P+ software (version 15.0.2.5959, Umetrics, Umea, Sweden).

(A) Permutation analysis plotting R2 and Q2 from 200 permutation tests in the OPLS-DA model. The y-axis shows R2 and Q2 and the x-axis shows the correlation coefficient of permuted and observed data. The two points on the right represent the observed R2 (green point) and Q2 (blue point). Cluster of points on the left represents 200 permuted R2s (green points) and Q2s (blue points). Dashed lines mark corresponding fitted regression lines for the observed and the permutated R2 and Q2; (B) Hotelling's T2 line plot. The plot displays the distance from the origin in the model plane (score space) for each selected observation. The plot shows the T2 calculated for the range of selected components. Red and orange horizontal dashed line denotes 99% and 95% CI level. Values larger than the 95% confidence limit are suspect, and values larger than the 99% confidence limit can be considered as serious; (C) DModX test plot. DModX is the distance of an observation in the training set to the X model plane or hyper plane. DModX is proportional to the residual standard deviation (RSD) of the X observation. Observations with a DModX twice as large as Dcrit (critical value of Dcrit) are moderate outliers.
